# Highly Parallel Droplet Dispensing Approach to Provide Homogeneous and Controllable Droplet Arrays for Diagnostic Test Manufacturing †

**DOI:** 10.3390/mi15070824

**Published:** 2024-06-26

**Authors:** Omid Rajabnia, Andreas Ernst, Nils Lass, Lutz Riegger, Roland Zengerle

**Affiliations:** 1 BioFluidix GmbH, Georges-Köhler-Allee 302, 79110 Freiburg, Germany; 2 Department of Microsystems Engineering (IMTEK), University of Freiburg, Georges-Köhler-Allee 103, 79110 Freiburg, Germany; 3 Hahn-Schickard, Georges-Köhler-Allee 103, 79110 Freiburg, Germany

**Keywords:** surface coating, biochips, highly parallel, droplet dispensing, homogeneous droplets, microfluidic

## Abstract

We introduce a novel approach for highly parallel droplet dispensing with precise control over the droplet parameters such as droplet volume, droplet velocity, etc. This approach facilitates the fabrication of homogeneous and precise thin layers with uniform coverage on defined small areas (e.g., a specific area of 1 × 1.4 mm^2^ in microfluidic channels or microwells). The presented approach ensures layer uniformity and high precision in X/Y extent and edge resolution, making it well suited for achieving precise and controlled coating for a variety of applications such as homogeneous coatings for lateral flow tests, ELISA plates, and biosensors for continuous glucose monitoring (CGM) devices. Our approach is based on direct liquid displacement employing a piston that is in direct contact with the liquid and an array of nozzles. Considering a variety of nozzle chip designs (i.e., varying nozzle diameter and pitch), we evaluated a multitude of parameters to derive general design rules for the nozzle chip design. Thus, we achieved a tunable droplet volume from 200 to 800 pL and droplet velocities from 0.5 to 2.5 m/s, applying a nozzle diameter of 50 μm and a nozzle pitch of 165 μm. The presented results showcase the versatility of the approach, offering precise dispensing capabilities.

## 1. Introduction

Surface coating processes play a pivotal role in industries ranging from electronics to biomedical devices such as biochips, modifying surface interaction with bioactive materials. Uniformity and optimal thickness are crucial for achieving full functionality of surface coatings [1]. Therefore, pursuing precision, efficiency, homogeneity, and scalability in manufacturing has driven the development of innovative techniques for small-scale and high-throughput diagnostic applications. This paper presents an innovative approach to highly parallel droplet dispensing [2] to fabricate homogeneous and precise thin layers for functionalizing defined small surfaces such as within microfluidic channels.

**Figure 1:**
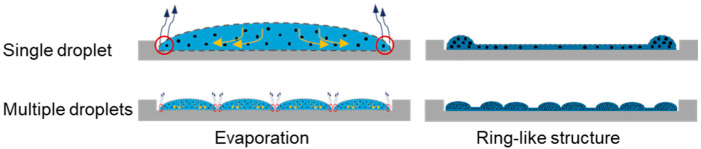
Illustrating a possible reduction in the coffee stain effect.

Common surface coating methods in diagnostic applications such as spray coating, inkjet printing [3], and microcontact printing often rely on single-nozzle, parallel-nozzle [4], and batch processes [5], which present various challenges. These methods frequently encounter issues like non-homogeneous liquid distribution on the surface, liquid handling limitations, material inefficiency, limited material adaptability, and varying coating precision and thickness. For instance, spray-coating methods face considerable challenges in achieving conformal coatings, managing the complexities of coating solution preparation, and coating specific areas on the substrate (spray-on-demand). Other methods, such as inkjet printing, also have limitations. One of the primary limitations of inkjet printing is the challenge of ensuring reliability and consistency, especially when using multiple nozzles [6]. This aspect can cause variations in droplet deposition that affect the quality and reproducibility of the coated surfaces. Moreover, the limitations on the fluids that can be printed are critical for additive manufacturing processes like inkjet printing [7]. The properties of the fluids used in inkjet printing, including viscosity, surface tension, and compatibility with the printing system, can impact the quality of the coatings and the overall manufacturing process in high-throughput settings. In contrast, the presented approach is distinguished by its ability to achieve a homogeneous and precise surface coating in a defined small surface area via highly parallel droplet dispensing incorporating array-on-demand (microarray) in a low volume range. It also offers customization capabilities in pattern design and nozzle size compared with other methods, like inkjet printing, which has a standard format design. Furthermore, this approach can reduce the coffee stain effect (Figure 1) and enhance the surface coating quality by depositing a multitude of smaller droplets. This decreases the impact of evaporation and capillary flow, which ensures the solutes within the microarray remain uniformly distributed, resulting in a more even and consistent deposition pattern. Thus, this method has the potential capability for homogeneous distribution of solids.

The new device can also be connected to a supply reservoir, where the pressure is meticulously controlled. This capability ensures a steady and adequate supply of material, effectively addressing the material supply limitations commonly encountered in high-throughput applications. The integration with a controlled supply reservoir allows continuous and uninterrupted dispensing, which is crucial for maintaining the high efficiency required in manufacturing environments.

In addition, the adaptability of the new device to various liquid properties extends its functionality to a broad spectrum of applications. The controlled environment provided by the device allows fine-tuning of the dispensing parameters to match the specific needs of different liquids. For instance, liquids with low surface tension can be handled without excessive spreading or droplet merging, while highly viscous liquids can be dispensed without clogging or inconsistent flow. This adaptability ensures that the device can be effectively used with a diverse range of fluids.

## 2. Materials and Methods

### 2.1. Principle, Design, and Fabrication

**Figure 2:**
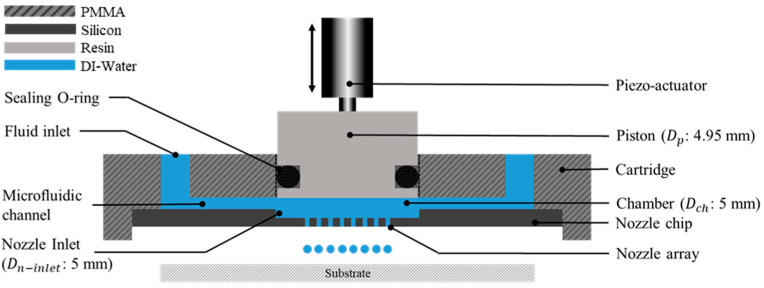
Dispensing device schematic.

The principle of the employed dispensing approach is based on direct liquid volume displacement for highly parallel droplet ejection through a nozzle array. The device comprises a cartridge designed with feeding channels directing the flow from the reservoir to the chamber. A piezo actuator, linked to a piston, facilitates the direct volume displacement within the cartridge chamber. Additionally, a nozzle chip has been incorporated for the controlled ejection of a droplet array positioned beneath the chamber. This arrangement effectively seals the microfluidic channels within the cartridge, as illustrated in Figure 2.

**Figure 3:**
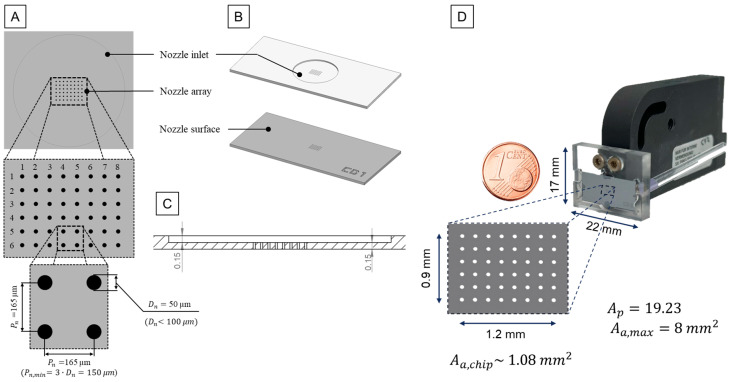
An example nozzle chip design according to the determined empirical design rules. (**A**) detail of the nozzle array design; (**B**) definition of the chip surfaces, including nozzle inlet and nozzle surface; (**C**) cross-sectional view of the nozzle chip indicating the nozzle inlet and nozzle height; (**D**) an example of the assembled cartridge and the actuator.

The cartridge is made of milled PMMA material and has been designed to contain two feeding channels with a height of 200 μm. The channels are connected to the circular chamber located in the center of the cartridge. Underneath the cartridge, a nozzle chip fabricated in silicon through deep reactive ion etching (DRIE) is attached to the cartridge, where the nozzle array is located beneath the chamber. In addition, a piston was fabricated in 3DM-Tough resin material using a Prusa sl1s printer (Prusa Research company, Prague, Czech Republic). The piston design included a cut-out for the placement of an O-ring to seal the chamber. This is positioned inside the chamber while being connected to the piezo actuator (a piezo stack actuator implemented in a special housing made by BioFluidix GmbH, Freiburg, Germany). The chamber’s volume is adjustable via the piston position, which was set to a height of 400 μm for the presented results. The nozzle chip is made from a 300 μm thick silicon wafer and has a nozzle inlet at a depth of 150 μm and through-etched nozzles (Figure 3C).

This paper introduces an empirical design rule for nozzle chips, specifying nozzle diameter, nozzle pitch, and array size relative to droplet volume and piston surface area. The underlying data were acquired through characterizing nozzle chips with different configurations and designs. We investigated nozzle diameters ranging from 40 to 200 μm and nozzle pitch to nozzle diameter ratios of 1 to 3.

Minimum nozzle pitch:

If $D_{n}$ < 100 µm:

(1) $$P_{n,min}=3\cdot D_{n}$$

If $D_{n}$ ≥ 100 µm:

(2) $$P_{n,min}=2\cdot D_{n}$$

where $D_{n}$ is nozzle diameter and $P_{n,min}$ is minimum nozzle pitch

Maximum array size in X and Y directions:

(3) $$A_{a,max}=\frac{1}{2.4}\cdot A_{P}$$

where $A_{P}$ is piston area (mm^2^) and $A_{a}$ is array area (mm^2^)

The nozzle surface was selectively coated with fluorosilane [8] using the vapor-phase deposition method to achieve a hydrophobic surface; in contrast, the nozzle inlet remained hydrophilic. A hydrophobic surface aids in the quick and clean tear-off of droplets from the nozzle tip because the reduced interaction between the fluids and the surface decreases surface tension effects. In contrast, hydrophilic surfaces, due to their high affinity for water and aqueous solutions, significantly enhance the wettability, enabling fluids to spread uniformly across microfluidic channels.

**Figure 4:**
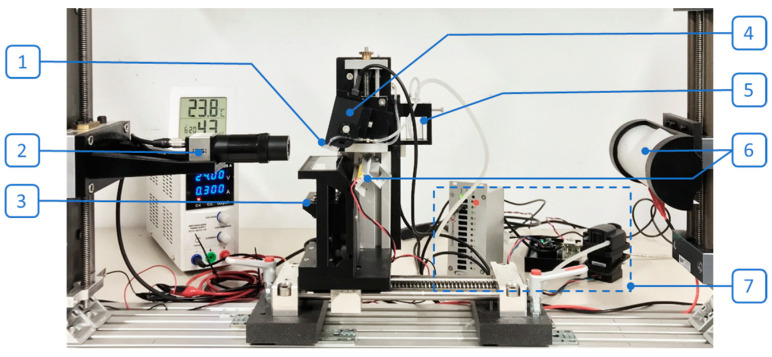
Experimental setup: (1) cartridge, (2) SmartDrop camera, (3) bottom-view camera, (4) actuator, (5) pressured reservoir, (6) illuminations, (7) control units including control electronics and a pump.

Our implemented experimental setup (Figure 4) allowed us to analyze the droplet tear-off, measure the droplet volume in flight using image processing [9], and visualize the spotted array on the substrate.

### 2.2. Operational Process and Experiment

The aim was to dispense highly parallel and homogeneous droplets within an array, which is essential for ensuring the homogeneity of a surface coating procedure. Consequently, maintaining stable operation is a fundamental necessity for the droplet dispensing process.

**Figure 5:**
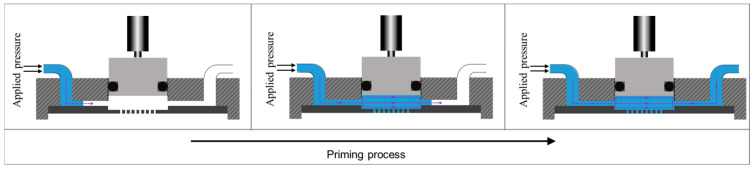
The priming process to wet the fluidic channels and chamber.

To achieve stable operation, a bubble-free priming process was established to ensure that the fluidic path, especially the chamber, was free of any air bubbles that could negatively impact the dispensing process, such as causing fluctuations in droplet volumes. In the priming process, the liquid flowed from one side of the feeding channel to the other side, while the other side end was open, to fully prime and wet the fluidic path and the chamber using a pressure setting of 0.5 mbar; see Figure 5. The priming pressure of 0.5 mbar, provided by a pressured reservoir connected to the feeding channel, as shown in Figure 4, was defined after the characterization of different pressures from 0.1 to 1 mbar with DI water for the priming process.

**Figure 6:**
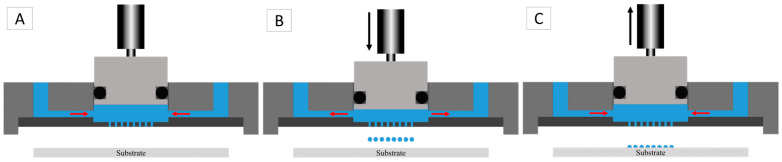
The schematic shows the displacement volume inside the chamber leading to the ejection of droplets from the nozzle array: (**A**) liquid pressure direction into the chamber before, during, and after the movement of the piston; (**B**) distribution of the displaced volume via downward movement of the piston to the inlet/outlet and ejected through the nozzles; and (**C**) refilling the chamber while the piston moves upwards.

To achieve droplet ejection through the nozzle array, a defined liquid volume was directly displaced by the piston inside the chamber without any interface, such as a membrane, air cushion, or a deformable medium (e.g., rubber). This movement was precisely controlled by a piezo-stack actuator that converted the amplitude and the speed of the piston directly into droplet volume and droplet speed (Figure 6).

Therefore, the applied energy was constrained within a specific range exceeding the surface energy of the liquid within the nozzles up to a point when satellite droplets appeared. The schematic in Figure 6 illustrates the mechanism of volume displacement within the chamber resulting in the ejection of droplets from a nozzle array. In step (A), pressure was applied to the liquid within the chamber throughout the piston’s cycle, maintaining a consistent baseline pressure before, during, and after the piston’s movement. This consistent pressure ensured the liquid was primed for displacement. In step (B), the downward movement of the piston displaced a specific volume of liquid. This displaced volume was distributed to the inlet/outlet and ejected through the nozzles, resulting in the formation of droplets. The controlled downward motion of the piston created the necessary force to push the liquid out through the nozzles uniformly. In step (C), as the piston moved back upwards, the chamber refilled with liquid under sustained pressure. This refilling process ensured the chamber was ready for the next cycle, maintaining a continuous and steady ejection of droplets. This cyclical process, driven by precise piston movements and consistent liquid pressure, ensured efficient and controlled droplet formation through the nozzle array.

## 3. Results and Discussion

This study evaluated various nozzle array configurations, exploring relationships between nozzle diameter, nozzle pitch, maximum array size, and droplet volume. The characterization of different nozzle chip configurations indicated two distinct regimes to calculate the minimum required nozzle pitch relative to the nozzle diameter (Figure 3). Formulas (1), (2), and (3) are derived from this characterization and provide practical insights for nozzle chip design.

**Figure 7:**
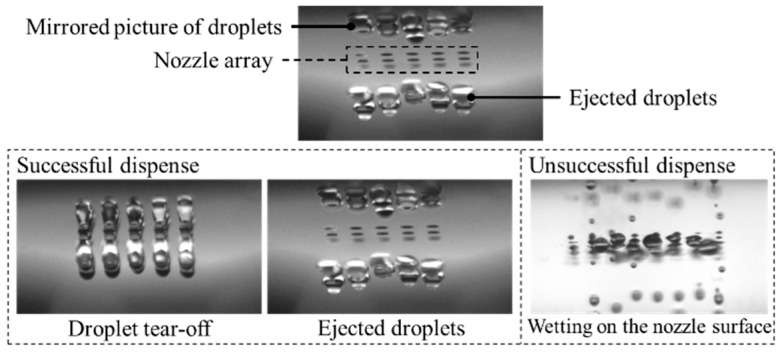
Successful and unsuccessful dispensing.

A nozzle chip with $P_{n}$ ≥ $P_{n,min}$ resulted in successful dispensing while $P_{n}$ < $P_{n,min}$ led to unsuccessful dispensing (Figure 7). Additionally, the issue with a smaller surface area between nozzles (smaller than the defined minimum nozzle pitch) in the context of hydrophobic coating may result in the compromised quality of the coating, and it becomes challenging to maintain a uniform and effective hydrophobic coating on the surface between the nozzles. Therefore, insufficient coverage could lead to areas with lower contact angles, especially around nozzles, demanding increased energy to overcome surface energy for droplet ejection. However, if the energy exceeds the optimal range, it can contribute to issues such as droplet merging, bulging, and wetting, ultimately resulting in unsuccessful dispensing.

**Figure 8:**
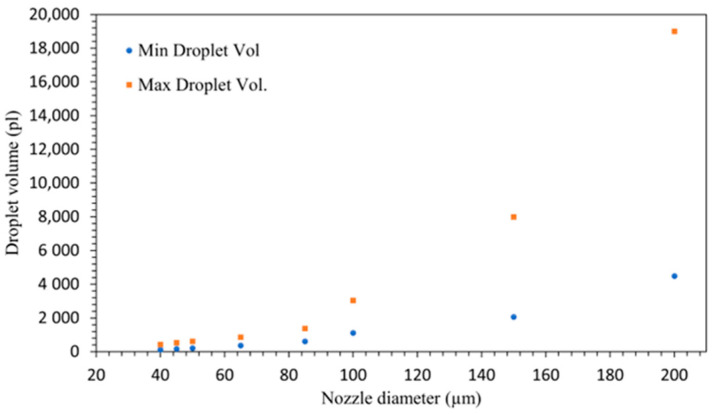
Measured minimum and maximum dispensed droplet volume according to the experimental results for nozzles between 40 and 200 µm.

The evaluation of droplet volumes over the experiment with different nozzle diameters ranging from 40 to 200 µm resulted in achievable dispensed droplet volumes from 140 pL to 20 nL, as shown in Figure 8.

Figure 8 illustrates the relationship between nozzle diameter and droplet volume, highlighting minimum droplet volumes ranging from approximately 140 pL to 4500 pL and maximum droplet volumes spanning from 400 pL to 19,500 pL. Both minimum and maximum droplet volumes increased with the increasing nozzle diameter. This suggests that larger nozzles produce larger droplets, which is consistent with the expectation that more fluid can pass through a larger aperture.

The relationship followed a nonlinear pattern of exponential growth, where the droplet volume was proportional to the nozzle diameter raised to an exponent. This indicates a more complex interaction between diameter and volume than a simple linear correlation. This behavior suggests that larger nozzle diameters allow more fluid to be ejected per unit of time, resulting in larger droplets. For small nozzle diameters such as 50 µm, both minimum and maximum droplet volumes were relatively low and showed minimal variation. The results imply that smaller nozzles (40–65 µm) are suitable for applications requiring precise control over smaller droplet volumes, albeit with a narrower adjustment range. These findings are also essential to ensure that the chosen nozzle diameters meet the precise droplet volume requirements necessary for high-precision droplet dispensing applications.

**Figure 9:**
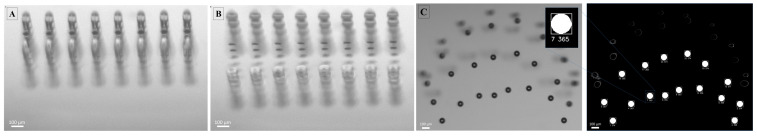
(**A**) droplets during tear-off; (**B**) droplets after tear-off; (**C**) parallel droplet volume measurement in flight.

**Figure 10:**
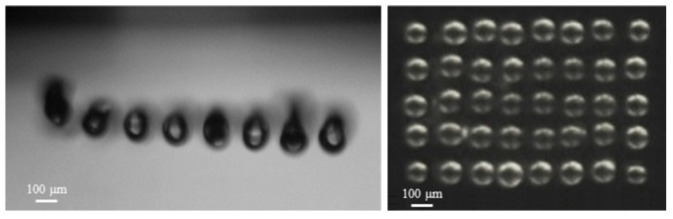
The dispensed droplet array in the fly (**Left**), and the spotted array on an objective slide (**Right**).

We utilized a specific nozzle chip configuration featuring a 50 µm nozzle diameter, a 165 µm nozzle pitch, and an 8 × 6 rectangular pattern to demonstrate droplet tear-off and droplet volume measurement in flight (Figure 9) and spotted an array on a microscopic slide made of PMMA with a contact angle of ~80° (Figure 10). Achievable droplet volumes ranged from 200 to 800 pL, dispensed with piezo strokes from 1 to 4 µm and stroke velocities from 20 to 45 µm/ms. The calculated coefficient of variation (CV) for droplet volume remained below 2.5% for a piezo stroke of 1.7 µm and stroke velocity of 30 µm/ms. This resulted in a dispensed mean droplet volume of 366 pL.

**Figure 11:**
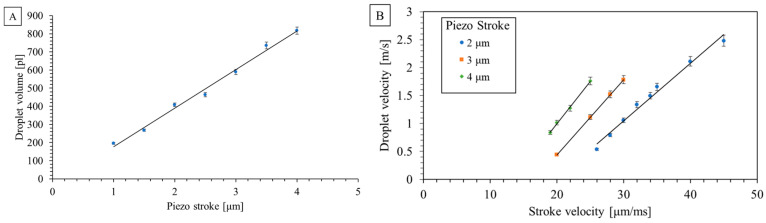
(**A**) measured dispensed droplet volume vs piezo stroke. (**B**) The effect of piezo actuator velocity on droplet velocity for three different piezo strokes.

The dispensed droplet volume was quantified as a function of the piezo stroke, shown in Figure 11A.

The R-squared value of 0.98 implies a high degree of linearity between droplet volume and piezo stroke, as referred to in Figure 11A. Experiments were conducted with three specific piezo strokes over different time intervals to analyze the relationship between the stroke velocity and droplet velocity, as shown in Figure 11B.

The direct linkage between the piston and droplet ejection implies an anticipated direct proportionality between droplet velocity and stroke velocity. The linearity of the droplet and stroke velocity relationship is shown in Figure 11B. The velocity measurements revealed that for every droplet volume, a specific speed range existed within which dispensed droplets remained stable without the appearance of satellite droplets. For instance, smaller droplets of 300 pL exhibited stable ejection within the range of 0.5 to 2.5 m/s, while larger droplets of 800 pL maintained stability between 0.8 and 1.8 m/s.

**Figure 12:**
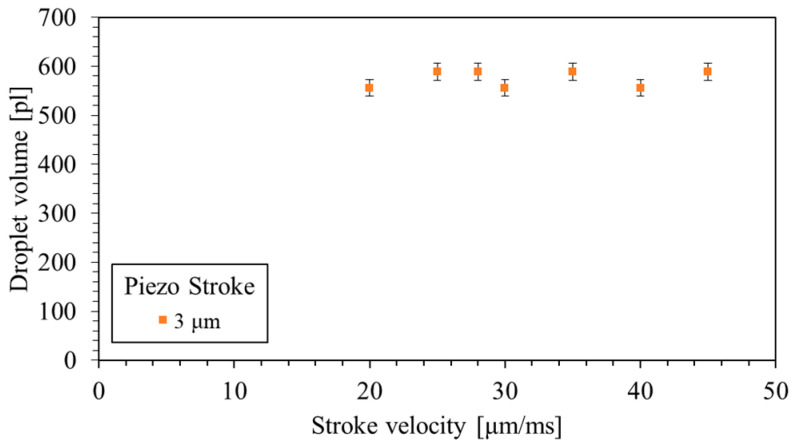
The effect of the stroke velocity on droplet volume at a constant 3 µm stroke. The error bars represent a 2.5% uncertainty in the measured droplet volume.

Furthermore, an experiment was conducted to evaluate the effect of stroke velocity on the droplet volume at the same piezo stroke. The results indicated that the stroke velocity had a minor impact on the droplet volume; see Figure 12.

Different water/glycerol mixtures were utilized as printing solutions to examine the impact of varying viscosity on droplet volume. The correlation between the weight percentage of glycerol and its viscosity is detailed in Table 1 [10]. Consistent with theoretical expectations, an increase in viscosity necessitated a higher energy supply from the piezo actuator to eject the droplet. This adjustment implies an increased minimum piezo stroke is required for stable droplet ejection with rising viscosity levels. Specifically, stable ejection commenced at a piezo stroke of 1 μm when using water as the printing solution. In contrast, a 50% glycerol/water mixture (viscosity of 6 mPa·s @ 20 °C) required a minimum piezo stroke of 3 μm to achieve stable dispensing.

**Figure 13:**
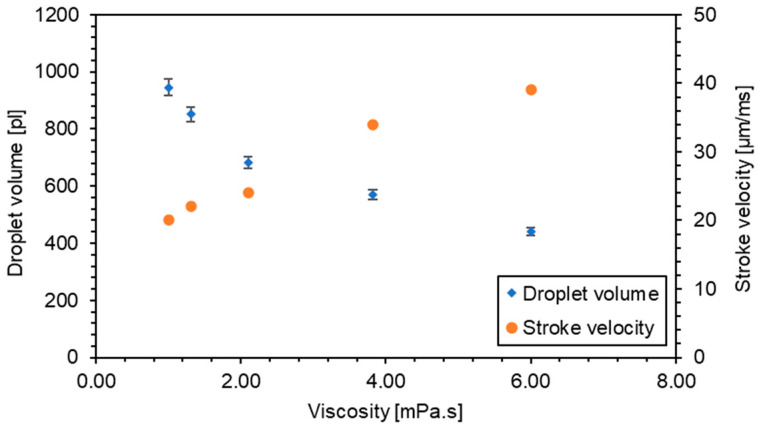
The effect of media viscosity on droplet volume and required stroke velocity at a constant piezo stroke of 3 µm.

Throughout the experimental trials, the piezo stroke was maintained at a constant 3 μm. To compensate for the increased viscosity, the velocity of the piezo was adjusted upwards to attain the necessary critical liquid velocity at the nozzle outlet, thereby ensuring successful droplet ejection. Conversely, for less viscous fluids, it is essential to reduce the piezo velocity to maintain stable operation and prevent the formation of satellite droplets. A notable reduction in droplet volume from 946 pL to 442 pL was observed when the viscosity increased from 1 mPa·s (water at 20 °C) to 6 mPa·s (50% glycerol solution at 20 °C), as depicted in Figure 13.

High-viscosity fluids have a greater resistance to flow due to stronger internal cohesion and frictional forces within the fluid. As viscosity increases, the fluid moves more sluggishly under the same actuation force (piezo stroke), which, in practical terms, means that less fluid can be pushed through the tiny nozzles compared with a less viscous fluid and correspondingly more fluid is pushed back into the reservoir. This results in smaller droplets being ejected for each actuation cycle; see Figure 13.

Furthermore, with increased viscosity, the flow characteristics through the nozzle change significantly. It becomes necessary to increase the piezo stroke velocity to achieve a similar droplet formation and ejection as seen with lower viscosity fluids. This adjustment helps overcome the increased fluid resistance and assists in achieving the critical velocity at the nozzle outlet necessary for droplet formation and ejection. On the other hand, by increasing the stroke velocity, the effective viscosity at the nozzle outlet is reduced, which can facilitate the ejection of the droplet. This phenomenon helps maintain droplet ejection efficiency but does not necessarily compensate fully for the increased viscosity, which is why smaller droplets are still observed.

Additionally, higher viscosity results in greater energy dissipation within the fluid due to internal friction. This requires not only a faster piezo stroke but potentially more forceful actuation to maintain a flow rate sufficient for proper droplet formation.

Surface tension and contact angle are factors that critically influence droplet dispensing through a nozzle array. Surface tension, resulting from cohesive forces between liquid molecules, affects droplet formation, detachment, and stability by minimizing the liquid’s surface area. The contact angle, influenced by surface tension and nozzle surface properties, determines the wettability of the surface, which is crucial for droplet ejection. Hydrophobic coatings on the nozzle increase the contact angle, reducing wettability and aiding in cleaner droplet detachment. This interaction ensures that droplets form discrete, uniform shapes essential for precise fluid delivery. Smaller nozzles require higher energy to overcome surface tension and form droplets, while larger nozzles can form larger droplets more easily. Fluids with adjusted surface tension, often modified with surfactants, can optimize droplet formation. Hydrophobic coatings maintain efficiency via preventing residual liquid build-up and clogging. Understanding and controlling surface tension and contact angle are essential for designing reliable droplet dispensing systems, ensuring consistent and accurate droplet ejection for various applications.

For a long-term experiment, a dispensing cycle was programmed to continuously dispense droplets over a 12-h period. Each cycle involved dispensing droplets four times at a frequency of 4 Hz, followed by a 15-s pause. At the end of the experiment, the array was printed on a substrate to show the quality of the printed array after long-term dispensing (see Figure 10). This programmed cycle was designed to simulate an example production line environment where the device dispensed droplets at four distinct positions, mimicking the movement and operational conditions of a manufacturing process. The inclusion of a pause time is crucial as it can be used to carry out further control of the coated surface, axis movement, or any other production or analysis process while the system is in a steady-state situation. Therefore, a pause time was programmed into the cycle can ensure that dispensing consistency could also be achieved after a steady-state situation. This approach helped in assessing the reliability and stability of the dispensing device, verifying its ability to maintain precise and consistent droplet formation and ejection throughout prolonged usage, essential for high-throughput manufacturing applications.

**Figure 14:**
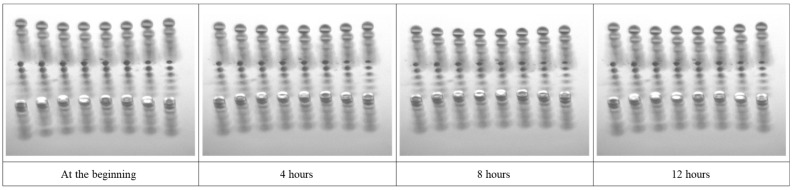
Long-term experiment depicting droplet dispensing in flight over a duration of 12 h.

Figure 14 illustrates the dispensed droplets in flight at different times, showing the nozzle surface and droplet dispensing behavior over a period of time. In total, the device dispensed droplets 10,800 times over a duration of 12 h, indicating the sustained behavior and stability of droplet formation with the specified configuration. 

## 4. Conclusions

In conclusion, this approach showcased a robust mechanism for the direct displacement of liquid volume, resulting in the precise and stable highly parallel dispensing of droplets onto a substrate.

This displacement of the liquid volume by the piston in the chamber, controlled by the piezo stack actuator, enabled direct control of the droplet ejection dynamics.

Experimental evaluations provided empirical design rules for the nozzle chips, defining relationships between nozzle diameter, pitch, and array size. This study identified successful dispensing conditions, outlined challenges, and devised formulas for the minimum required nozzle pitch relative to nozzle diameter, offering practical guidelines for successful dispensing.

The characterization of nozzle chip configurations highlighted the device’s versatility, allowing dispensing volumes ranging from 140 pL to 20 nL with different nozzle diameters from 40 to 200 µm. The specific configuration with a 50 µm nozzle diameter, 165 µm nozzle pitch, and an 8 × 6 rectangular pattern demonstrated successful dispensing in the range of 1 to 4 μm and 20 to 45 μm/ms for piezo stroke and piezo stroke velocity, respectively. Furthermore, specific dispensing parameters used to indicate successful droplet tear-off, droplet volume measurement, and spotted array on the substrate resulted in a mean droplet volume with a CV of 2.5% in an array of droplets.

Quantifying dispensed droplet volume as a function of the piston stroke revealed a consistent linear relationship, indicating precise volume control. The experiments analyzing the relationship between piezo actuator velocity and droplet velocity underscored the device’s capability to maintain stability within specific velocity ranges for different droplet volumes.

Moreover, the observed decrease in droplet volume with increased viscosity, with constant piezo stroke displacement, was mainly due to the increased resistance to flow within the tiny nozzles and changes in the fluid’s physical behavior under stress. Increasing the stroke velocity helped mitigate these effects somewhat by reducing effective viscosity at the point of ejection and facilitating droplet formation, but it did not fully offset the intrinsic properties of the higher-viscosity fluid. This interplay between viscosity, piezo actuation, and droplet formation dynamics is crucial in optimizing dispensing systems for various applications.

The programmed dispensing cycle, simulating an example production line environment, effectively demonstrated the device’s capability to consistently dispense droplets over an extended 12-h period. These findings underscore the device’s suitability for continuous operation in industrial settings.

Overall, this approach addresses several key aspects and challenges of the surface coating technology for diagnostic test manufacturing. In addition, our approach demonstrates robustness through concurrently printing droplets onto the surface in the desired format, as opposed to individual channel printing, thereby simplifying controllability. Furthermore, our approach offers customization capabilities in pattern design and nozzle size, enabling compatibility with the cartridge and actual fluidic pathway configurations. Additional research will be conducted to assess all system variables and optimized parameters associated with the specified liquid. Further investigation will be undertaken to analyze the homogeneity of the dispensed layer and its thickness on the specified surface.

## Figures and Tables

**Table 1:** The viscosity of glycerin solution in DI water @ 20 °C.

Glycerol Percent Weight [%]	0	10	25	40	50
Viscosity [mPa·s]	1.005	1.310	2.100	3.720	6.000

## Data Availability

Data are contained within the article. Further inquiries can be directed to the corresponding author.
